# Understanding the hydrological and landscape connectivity of lakes

**DOI:** 10.1007/s10980-025-02153-6

**Published:** 2025-07-03

**Authors:** P. Taylor, L. Carvalho, D. Chapman, A. Law, C. Miller, M. Scott, G. Siriwardena, S. J. Thackeray, C. Ward, C. Wilkie, N. Willby

**Affiliations:** 1 UK Centre for Ecology & Hydrology (UKCEH), Bush Estate, Penicuik, EH26 0QB UK; 2 UK Centre for Ecology & Hydrology (UKCEH), Library Avenue, Bailrigg, Lancaster, LA1 4AP UK; 3 Norwegian Institute for Water Research (NIVA), Økernveien 94, 0579 Oslo, Norway; 4 Biological and Environmental Sciences, University of Stirling, Stirling, FK9 4LA UK; 5 School of Mathematics and Statistics, University of Glasgow, Glasgow, G12 8QQ UK; 6 British Trust for Ornithology (BTO), The Nunnery, Thetford, Norfolk, IP24 2PU UK

**Keywords:** Freshwater, Hydrological connectivity, Lakes, Lake catchments, Ponds, Rivers

## Abstract

**Context:**

Connectivity is a key property of water, enabling the flow of energy, material and individuals within and between sites. Climate and land use changes can profoundly modify connectivity, yet few studies have quantified the patterns in connectivity among lakes at national scales.

**Objectives:**

Our objectives were: i) to examine relationships between a broad range of lake connectivity metrics, ii) to evaluate how lake connectivity varies nationally, regionally and in relation to land cover.

**Methods:**

We calculated hundreds of metrics of freshwater connectivity for all lakes in Great Britain > 1 ha (n = 10,095), quantifying connectedness in their catchments and surrounding landscape. Patterns of metrics, as well as their correlations and inter-connectedness, were examined at multiple scales.

**Results:**

Strong correlations existed within groups of metrics for lake, pond and river connectivity. However, both pond and river metrics varied independently of lake metrics. The most and least urban river basin districts showed noticeable differences in metric correlation. Lake area, pond count and river length in catchments were selected as a core set of connectivity metrics, which explain most of the variation across national and regional scales.

**Conclusions:**

Connectivity metrics can be synthesised to core groups that are easily calculated and effectively account for lake, pond and river connectivity. From a landscape management perspective, hydrological connectivity was highest *per unit area* in the zone nearest the lake. When interpreting ecological responses, the connectivity metric within each core group can be selected based on suitability and data availability. The minimum set of three metrics is recommended to support comparative, global studies.

## Introduction

Connectivity is a fundamental component of all ecosystems. In ecology, it is the state of an ecosystem which describes how energy, materials and species move around a landscape, both within and among habitats and though space and time. Our ecosystems are becoming increasingly fragmented through changes in land use, loss of habitat quality and quantity and our changing climate (Dudgeon 2019). Connectivity is crucial for functioning and resilient ecosystems. Therefore, increasing or maintaining connectivity within and amongst ecosystems is generally viewed as a positive goal for habitat management and conservation globally (Lawton 2010).

Freshwater habitats across landscapes are connected through flows of species and materials. These connections may be through transfer across land, with lateral connectivity to the adjacent riparian zone / floodplains being particularly important (Amoros & Bornette 2002); through species or their propagules in air (Lovas-Kiss et al. 2020); or hydrologically, by a temporary or permanent flow of water between sites through which material, species or their propagules can disperse (Fergus et al. 2017). Freshwater ecosystems can also be connected over longer distances through dispersal via the movement of humans or other animal vectors, such as birds, (Navarro-Ramos, 2022; Chapman et al. 2020). The impacts of connectivity pathways upon freshwater biota will also differ with respect to species dispersal traits (e.g. the ability of species to disperse across land, up or down stream or by air over different distances) (Heino et al. 2015; Sarremejane et al. 2017; Chapman et al. 2020).

Understanding landscape and hydrological connectivity is fundamentally important for managing and restoring freshwaters, where hydrological connections provide a pathway for species dispersal, including migrations of fish species of high economic importance (e.g. salmon, eel, sturgeon). Seasonal changes in connectivity also include natural cycles of flooding, which bring pulses of nutrients and organic matter from surrounding land, to stimulate production within freshwaters (Drake et al. 2021). Removing barriers to fish migration, such as dams, and reconnecting rivers to their floodplains are two current measures being widely implemented globally to restore the connectivity of freshwater (Kemp & O’Hanley 2010; Opperman et al. 2010) a principle enshrined in biodiversity policy targets, such as the UN Kunming-Montreal Global Biodiversity Framework agreement (targets 8 & 11) (UNEP, 2022) and the EU Nature Restoration Regulation (European Union, 2024). The importance of connectivity has also been highlighted for future biodiversity policy targets (van Rees et al. 2021).

Despite this, freshwater connectivity is rarely measured or analysed across broad landscapes and there is very limited understanding of how connectivity varies across different landscapes and land-uses. One exception is analysis of lakes across the north-east USA (Fergus et al. 2017) with evidence showing the dependence of landscape-scale ecology on this connectivity and the growing need for approaches on a macro-ecological scale (Epting et al. 2018). Macroscale connectivity metrics have been shown as useful predictors of water quality (Soranno et al. 2017), concentrations of nutrients (Lapierre et al. 2018; Wagner & Schliep 2018), dissolved organic matter (Hosen et al. 2018), and chlorophyll *a* (Filstrup et al. 2018). And more generally, connectivity metrics have been used to support better understanding and management of lake ecosystems (Hill et al. 2018) and ecological dispersal (Borthagaray et al. 2023).

However, increasing connectivity of freshwaters can also have detrimental effects on freshwater biodiversity and ecosystem functioning through the spread of stressors such as sediments, pollutants (Ormerod et al. 2010) and the movement of invasive non-native species and fish parasites (Chapman et al. 2020). Therefore, in some situations it may not be appropriate to restore connectivity until these stressors are reduced or mitigated. There is, therefore, a need to better understand connectivity of freshwaters to balance the negative against positive effects on freshwater habitats and their biodiversity. The connectivity of freshwater lakes is particularly poorly studied, despite their global distribution and importance. These ecosystems can be isolated “islands” in the landscape or well-connected to other lakes through a river network (Fergus et al. 2017). There is a clear need in freshwater research and management to determine these different components of connectivity and understand how they relate to each other and to freshwater stressors (e.g. agricultural and urban land use). In this paper, we identify and quantify a suite of freshwater connectivity metrics for lakes, which can be used to compare and characterize the connectivity of freshwaters across different landscape scales and to support improved understanding of the influence of connectivity on ecological processes. Our objectives are to establish connectivity groupings (correlations or independence among metrics) to then assess which minimal set of metrics represent the complex variation in freshwater connectivity in the most parsimonious way and establish whether different aspects of connectivity vary regionally.

In this paper, we address the following questions:

1. How are different lake connectivity metrics related to each other?
2. Do connectivity metrics related to different habitat types (lakes, rivers and ponds) provide similar or unique information on the overall connectedness of freshwaters in the landscape to lake ecosystems?
3. Do relationships among lake connectivity metrics differ spatially, and depend on landscape features such as land cover?

We hypothesise that correlations among connectivity metrics will depend upon the freshwater habitat types that they relate to, and that metrics will vary spatially at a regional scale driven by differences in land use.

## Methods

**Fig. 1:**
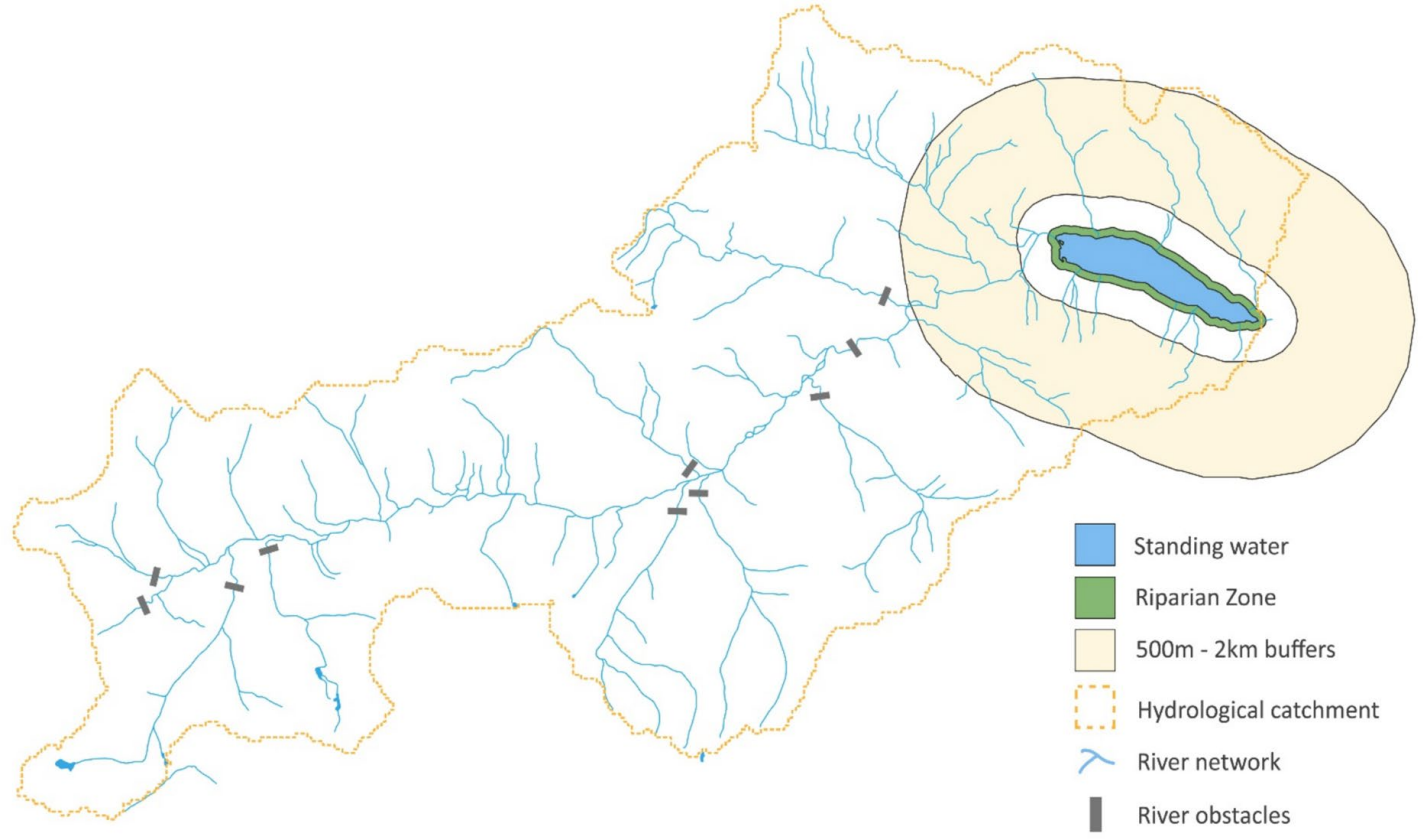
Connectivity of freshwaters via catchment hydrology, landscape and dispersal vectors, using Loch Beannacharain as an example

Using data from the UK Lakes Database (Hughes et al. 2004) and a 1:50,000 digital river network of Great Britain (Moore et al. 1994), the varying connectedness of lakes > 1 ha was calculated, mirroring, as much as practical, the approach taken by Fergus et al. (2017) in the United States. Lake polygons (adapted from OS PANORAMA data) and catchment polygons (hydrological watersheds delineated from a 50 m flow grid) were supplemented by creating lake buffer datasets in a GIS. We then calculated core metrics for two broad classes of connectivity ( Fig. 1 & Table 1):

- Hydrological Connectivity (metrics quantified within the lake catchments)
- Landscape Connectivity (metrics quantified with buffers of increasing distance from the lake perimeter), including lateral connectivity with the shoreline and riparian zone.

**Table 1:** List of calculated hydrological / landscape connectivity metrics and proxies of stress (obstacles and aggregated land cover classes)

Hydrological	Landscape
Mean slope in catchment (degrees) Mean elevation in catchment (m)	Mean slope in buffer (degrees) Mean elevation in buffer (m)
Lake area in catchment (ha)	Lake area in buffer (ha)
Pond area in catchment (ha)	Pond area in buffer (ha)
Lakes—Perimeter in catchment (m)	Lakes—Perimeter in buffer (m)
Ponds—Perimeter in catchment (m)	Ponds—Perimeter in buffer (m)
Lakes—Count in catchment	Lakes—Count in buffer
Ponds—Count in catchment	Ponds—Count in buffer
Rivers—length in catchment (m)	Rivers—length in buffer (m)
Canals—length in catchment (m)	Canals—length in buffer (m)
Strahler 1—length in catchment (m)	Strahler 1—length in buffer (m)
Strahler 2—length in catchment (m)	Strahler 2—length in buffer (m)
Strahler 3—length in catchment (m)	Strahler 3—length in buffer (m)
Strahler 4 +—length in catchment (m)	Strahler 4 +—length in buffer (m)
*Obstacles—Count in catchment*	*Obstacles—Count in buffer*
*LCM2007 – Agricultural land cover (ha)* *LCM2007 – Urban land cover (ha)*	*LCM2007 – Agricultural land cover (ha)* *LCM2007 – Urban land cover (ha)*

**Fig. 2:**
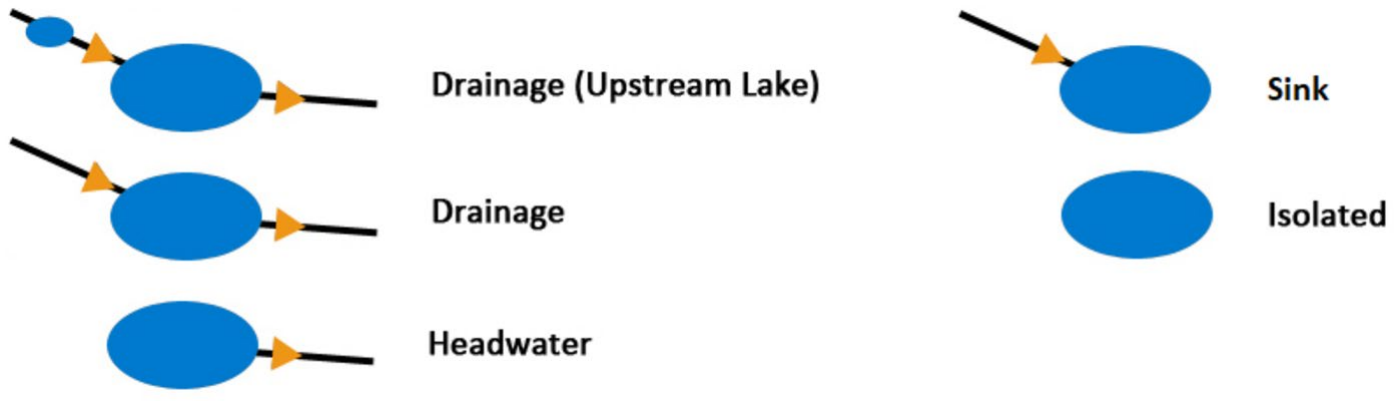
Lake connectedness categories, expanded from Fergus et al. (2017). Drainage (Upstream Lake): stream inlets and outlets and at least one upstream lake; Drainage: stream inlets and outlets; Headwater: only stream outlets; Sink: only steam inlets; Isolated: no stream inlets or outlets

A high-level metric of connectedness type was also calculated ( Fig. 2). Different types of connectivity may vary in their importance to different ecological impacts, e.g. hydrological connectivity metrics may impact more on obligately aquatic species, while landscape connectivity may impact more on species with terrestrial or aerial life stages. Therefore, it is important to consider both hydrological and landscape connectivity metrics. All metrics in Table 1 were then converted to a per area calculation to allow comparison between sites and their catchments and landscape buffers. It is worth noting that no distinction was made between lakes and reservoirs, so the terms ‘lake’ and ‘pond’ used throughout will also include any waterbodies used for water supply. Ponds represent waterbodies with an area < 1 ha, as set out in Maberly et al. (2024). For rivers, total length was provided in addition to a breakdown of that total length by stream order, represented by Strahler numbers. Canals were defined as lentic, linear, artificial waterbodies (Law et al. 2024).

### Data preparation

To calculate the connectivity metrics, datasets were first collated from the UK Lakes Portal, UK digital river network, OS MasterMap Water, River Obstacles (with some complementary data being provided by agencies in the United Kingdom) and Land Cover Map (LCM) 2007. As a proxy for stressors that lakes are exposed to, the LCM land cover data were used: LCM2007 classes 3 (‘Arable and Horticulture’) and 4 (‘Improved Grassland’) were combined into an agricultural land cover layer, whilst classes 22 (Urban) and 23 (Suburban) were combined into an urban land cover layer. Strahler stream order numbers were pre-calculated in the digital river network. A higher Strahler order number represents a more complex downstream tributary system, with a maximum classification of eighth order streams present in the United Kingdom. With orders over 4 representing < 14% or river segments in the United Kingdom, these were grouped into a 4 + category.

### Hydrological connectivity

We have focused on metrics of upstream hydrological connectivity, including those of river length and counts, area and perimeter of lakes and ponds in the upstream catchment ( Table 1), and all catchment metrics were calculated without their component lake i.e. a lake where the metric ‘Lakes—Count in catchment’ = 0, has only itself in its catchment. Obstacles or barriers to this hydrological connectivity were also counted, including weirs or waterfalls that may not act as complete barriers to all species or material moving downstream, but are likely to be influential to upstream movements of aquatic biota. Ponds are often hydrologically isolated on the surface, with small or often no catchments, so will have zero values for hydrological connectivity metrics when classed as a focal waterbody. However, they can still have groundwater connections that allow sub-surface transfer of nutrients and influence freshwater connectivity through aerial dispersal and acting as sources of biodiversity.

### Landscape connectivity

Different organism groups, and species within groups, have varying dispersal abilities across land and in the air. As a result, the appropriate scale of landscape connectivity will depend upon species dispersal traits. In the absence of a priori information on the optimal spatial scales to capture such dispersal limitation for a wide range of species, we have, therefore, calculated metrics of landscape connectivity for several, increasing distances of buffer zones from each waterbody: 100 m (a proxy for the riparian zone), 500 m, 1 km, 1.5 km and 2 km ( Fig. 1), where the 100 m and 500 m buffers mirror the approach of Soranno et al. (2017), with three further landscape buffers added to test connectivity at a greater landscape distance. From Fig. 1 it is clear that the higher buffer distances will also include greater area downstream of the lake catchment.

For each landscape buffer zone, metrics include measures of river length and counts, area or perimeter of lakes or ponds within the buffer distance from the waterbody shoreline, irrespective of whether they are in the hydrological catchment (Fig. 1; Table 1). Metrics of landscape connectivity will overlap with metrics of hydrological connectivity, however; in our relatively small buffer zones (up to 2 km), the buffer area will tend to be smaller than the hydrological catchment. Our metrics of landscape connectivity also include land downstream of the waterbody; as the buffer distance increases, this area outside the hydrological catchment increases proportionally compared with the area within the hydrological catchment (Fig. 1).

In addition to calculating the catchment and landscape connectivity metrics, we assigned each lake to one of five connectedness categories, initially defined by Fergus et al. (2017) and expanded here to include lakes with inflows but no (recorded) outflows ( Fig. 2), based upon the presence or absence of upstream and downstream hydrological linkages. These high-level categories of lake connectivity allow easy comparison across the two studies and beyond. One distinction was that the study in the US set a minimum lake area of 4 hectares, and a minimum area of a defined upstream lake at 10 hectares. To better suit our smaller scale study and the smaller lakes in the United Kingdom, both these values were set to 1 hectare – an area often used as the minimum for the definition of a ‘lake’ (Maberly et al. 2024).

### Data generation, exploration & statistical analysis

Data were analysed at two scales; national and river basin districts (RBDs) set out by the Water Framework Directive (EEA, 2019). For the national scale analysis, spatial data from the UK Lakes Portal (Taylor 2021) were used for all lakes and catchments, with the majority of lakes originally sourced from OS open data, and catchments delineated using 50 m flow direction and flow accumulation grids – derived from a digital elevation model (DEM) and river network. A quality assurance process was then performed on the lakes and catchments: for the lake and pond polygons, any that appeared erroneous in comparison to a satellite photo or map, represented saline or brackish water, or were waterbodies that no longer exist, were removed. For the catchments, a manual QA process was undertaken to remove erroneous delineations where lake outflows have likely been misaligned to the flow grid when the catchments were calculated – resulting in catchments that are vastly larger than the lake itself, which would be unusual in a natural system. Due to very high error rates for catchments with a catchment-to-lake area ratio (CLR) > 350, all were removed, then a sample of catchments below this value were assessed individually. Due to errors increasing above a threshold CLR value of 100, it was decided this limit should be applied across all lakes and all those whose catchments had a CLR value > 100 were removed from the analysis.

In total, 12 RBDs have been designated by regulatory authorities in Great Britain—Anglian, Dee, Humber, North West, Northumbria, Scotland, Severn, Solway Tweed, South East, South West, Thames, Western Wales ( Fig. 7). These regions were used to test if connectivity metrics differed between landscapes with disparate land uses. A large number of connectivity metrics were initially generated with no a priori assumptions regarding potential explanatory power or their inter-relatedness.

To better understand patterns in the correlations among metrics, and identify which metrics effectively *capture* most of the connectivity variation at the national scale and across regions, further analyses were carried out for each of the 12 RBDs. These differ in urban / agricultural land cover and geological features that we would expect to impact (types of) connectivity across the country and are therefore likely to affect how the connectivity metrics are correlated. Due to the limited number of lakes in the dataset for Northern Ireland, these were not analysed at this scale, but did contribute to the Great Britain-scale analysis.

Principal Components Analysis (PCA) was used to reduce the dimensionality of the multivariate connectivity metric data set and to identify a subset of metrics that could parsimoniously explain most of the variation in connectedness amongst sites, with a view to simplifying their use in further statistical analyses of biodiversity and ecosystem function. As it is important to consider whether variation in connectivity differs across geographical scales in Great Britain, the PCA was performed at different geographical scales (national and within RBDs). Finally, we examined how RBDs differed in terms of key connectivity metrics, through scatterplots showing means and standard deviations within each RBD. Data analyses and visualisations were performed in R 4.0 (R Core Team 2020) using the packages sf (Pebesma 2018), raster (Hijmans 2020), fasterize (Ross 2020), cluster (Maechler et al. 2020) and ggplot2 (Wickham 2009), and in QGIS (QGIS Development Team 2024). Further statistical analyses were conducted using R version 4.2.1 (R Core Team 2022) with the packages FactoMineR (Lê et al. 2008), factoextra (Kassambara & Mundt 2020) and ggplot2 (Wickham 2009).

Finally, to allow a trend analysis of hydrological connectivity metrics against buffer distance, the buffer metrics were log-transformed and scaled (by subtracting the mean then dividing by the standard deviation) as these values were not normally distributed and were zero-inflated. This made relative errors equal across metrics and buffer scales and comparisons across spatial scales possible.

## Results

### Hydrological connectivity

The lake / catchment quality assurance process produced a dataset of 10,095 lakes with defined catchments across Great Britain. Buffers were applied to all lake polygons (in the United Kingdom, not just Great Britain) for the assessment of landscape connectivity – at 100 m, 500 m, 1 km, 1.5 km and 2 km distances from the lake perimeter.

**Fig. 3:**
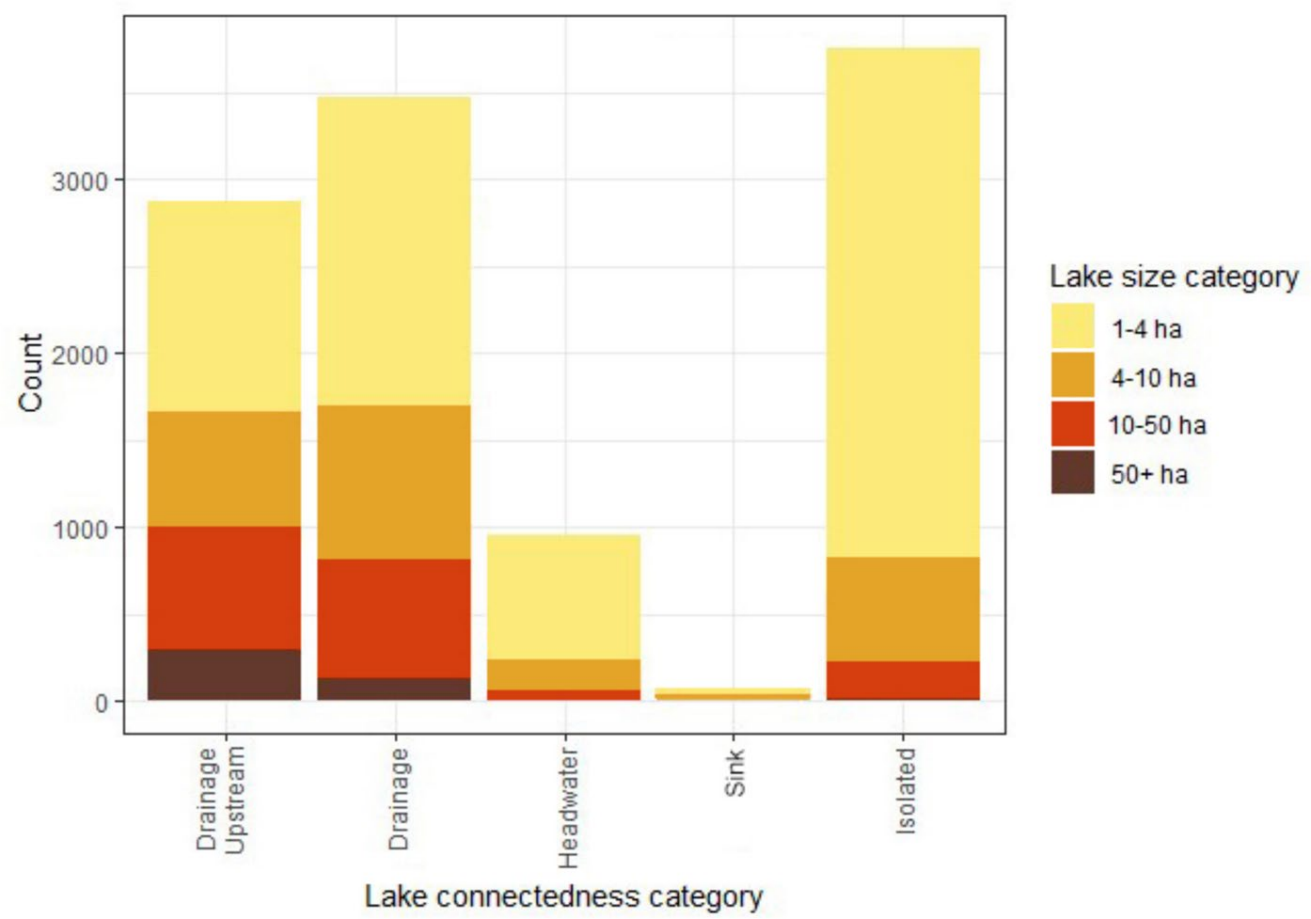
Lake counts and size classes of different lake connectedness categories

Hydrologically connected lakes out-numbered isolated lakes by nearly 2:1, although hydrologically isolated systems were the most numerous type for the smallest lake size category, and many may be considered as ponds (Richardson et al. 2022) ( Fig. 3). The larger lake classes (> 10 ha) were generally well connected to their landscape by drainage systems which had upstream lakes. In the United Kingdom, sink systems are rare, comprising only 1% of lakes.

**Fig. 4:**
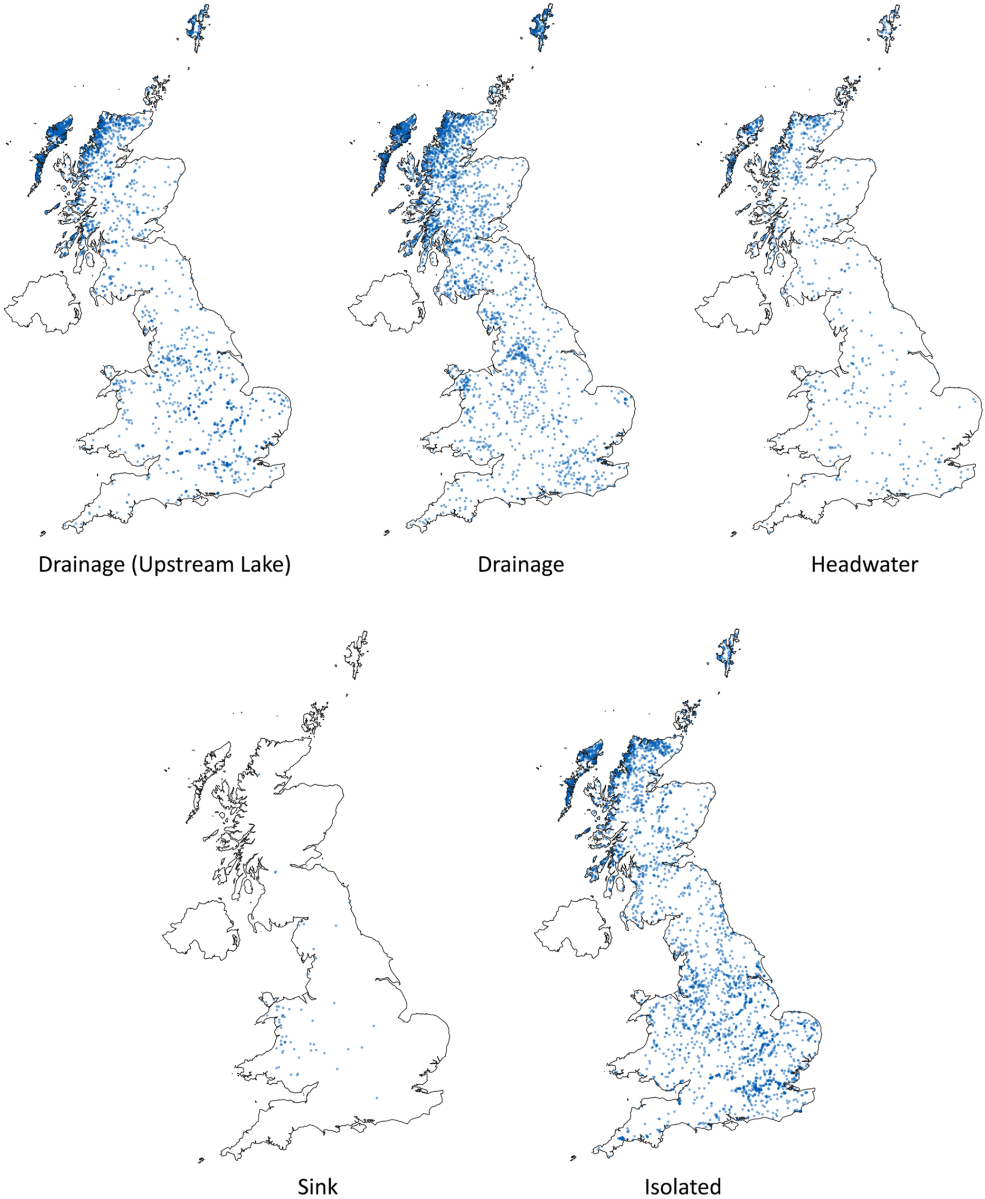
Geographic distribution of lakes based on connectedness type

Due in part to geological history (much of the northern two thirds of the area was glaciated), as well as urbanisation and agricultural patterns, regional differences in connectivity are observed. Fig. 4 shows the geographic distribution of the five types of lake connectedness as well as giving an overview of lake distribution across Great Britain. There are no clear spatial patterns of connectedness types across Great Britain, with ‘drainage’ and ‘headwater’ lakes having a broad distribution, generally following the distribution pattern of lakes in general (highest density in the north and west of Scotland). ‘Isolated’ lakes are more evenly distributed, occurring relatively more frequently in areas with lower lake density (mainly England), whereas ‘sink’ lakes are most common in Wales, although they are by far the least frequently occurring type.

**Fig. 5:**
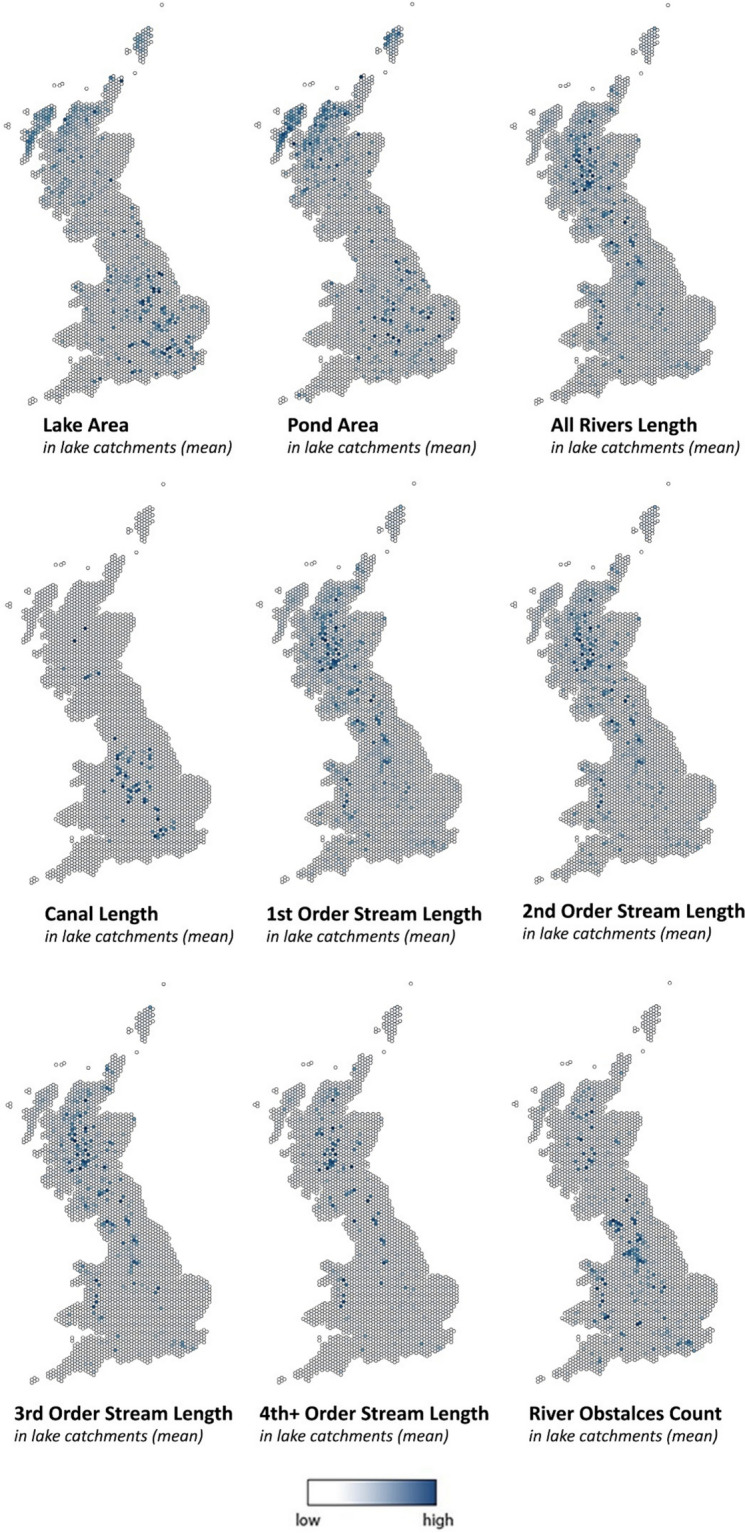
Equilateral hexagon (short diagonal = 10 km) polygon maps showing mean lake area, pond area, all rivers length, canal length, lengths for 4 different Strahler stream order categories, and river obstacle counts – all represented as mean values per catchment(s), with data/scales normalised from 0 (low) to 1 (high) and graduated using natural breaks

 Figure 5 shows the geographic distribution of lakes and ponds (by area), all rivers and 4 stream order categories (by length), and river obstacles (count) – all represented as mean values within lake catchments for equal area hexagons (short diagonal = 10 km) across Great Britain. Catchments with a high percentage of lake/pond area are concentrated in northwest Scotland and south-central England, whereas catchments with a large number of rivers exist mostly down the spine of the country and central Wales. Catchments with the most river obstacles are clustered in inland England and are aligned with areas of high population / urbanisation, whereas catchments with the most canals are mainly in the former industrial heartlands of the country.

### Landscape connectivity

Table 2 shows a range of connectivity metrics and how they vary across spatial scales, including mean values and standard deviation. For most values, the standard deviation is as large, if not larger, than the mean. Even across a small geographical extent, there is considerable variability across connectivity metrics.

**Table 2:** Average and standard deviation (sd) values for connectivity metrics and stressors of lakes in the United Kingdom, where riparian zone = 100 m buffer

	catchment—mean	*catchment – sd*	riparian zone—mean	*riparian zone—sd*	500 m buffer—mean	*500 m buffer—sd*	1 km buffer—mean	*1 km buffer—sd*	1.5 km buffer—mean	*1.5 km buffer—sd*	2 km buffer—mean	*2 km buffer—sd*
Mean elevation (m)	170	*168*	149	*158*	151	*157*	150	*152*	149	*147*	148	*142*
Mean slope (degrees)	4.8	*4.6*	3.7	*3.4*	4.7	*4.4*	5	*4.5*	5.2	*4.5*	5.3	*4.4*
Lake area %	1.7	*4.9*	1.1	*3.9*	2.9	*5.9*	3	*5.5*	2.9	*5.2*	2.5	*4.8*
Pond area %	0.58	*1.83*	1.14	*2.73*	0.66	*1.03*	0.46	*0.69*	0.38	*0.56*	0.31	*0.5*
Rivers—length (m/km^2^)	1160	*1460*	2127	*2136*	1589	*1044*	1534	*807*	1504	*724*	1478	*683*
Canals—length (m/km^2^)	14.3	*232.6*	20	*278.9*	16.8	*134.6*	15.1	*95.2*	14.1	*77.8*	13.6	*67.4*
Strahler 1—length (m/km^2^)	925	*1228*	1321	*1559*	871	*673*	826	*528*	804	*473*	788	*444*
Strahler 2—length (m/km^2^)	231	*575*	490	*1199*	410	*507*	393	*330*	382	*262*	370	*225*
Strahler 3—length (m/km^2^)	46	*269*	196	*859*	177	*403*	181	*273*	182	*210*	182	*171*
Strahler 4 +—length (m/km^2^)	23	*254*	167	*837*	156	*450*	151	*325*	150	*263*	149	*224*
Lakes—Perimeter (m/km^2^)	444	*1251*	451	*1372*	683	*1193*	616	*1015*	558	*906*	541	*881*
Ponds—Perimeter (m/km^2^)	394	*1119*	842	*1907*	463	*726*	322	*493*	266	*404*	235	*353*
Lakes—Count/km^2^	1.19	*6.19*	2.44	*6.48*	0.93	*1.43*	0.61	*0.87*	0.48	*0.68*	0.52	*1.23*
Ponds—Count/km^2^	2.4	*8.3*	6.2	*13.4*	2.1	*3.4*	1.4	*2.2*	1.1	*1.8*	1	*1.5*
LCM2007—Agricultural %	19.5	*30.3*	23.5	*32.8*	27.6	*34.1*	28.4	*34.1*	28.8	*33.9*	29	*33.8*
LCM2007—Urban %	4.2	*14.6*	3.4	*12.5*	4.4	*13.3*	4.8	*13.4*	5	*13.2*	5.1	*12.9*
Obstacles—Count/km^2^	0.07	*0.89*	0.7	*4.99*	0.23	*1.07*	0.16	*0.52*	0.14	*0.36*	0.13	*0.3*

**Fig. 6:**
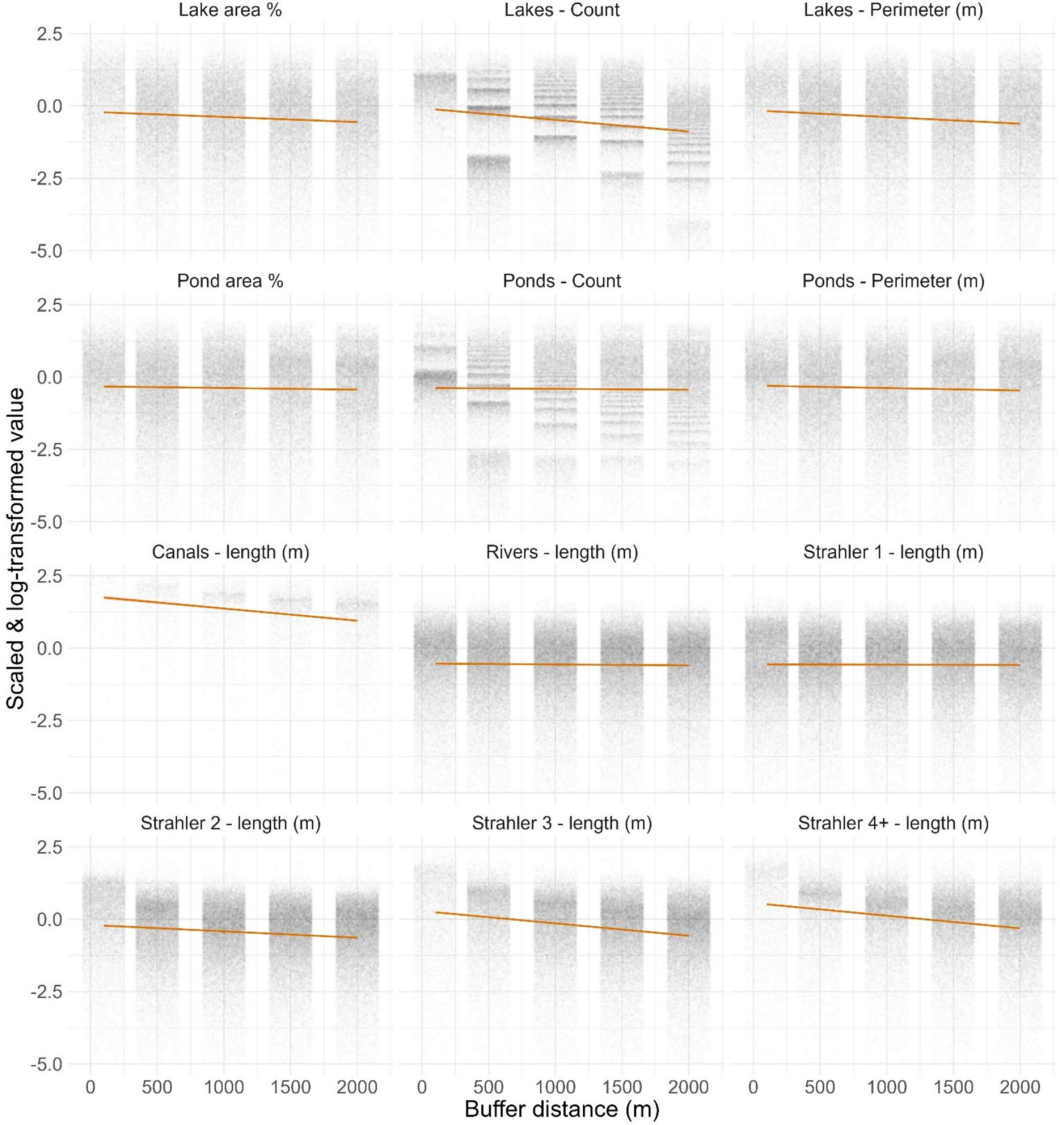
Trends in hydrological connectivity metrics as buffer distance increases. Values are scaled and log-transformed. The y-axis is limited to -5–2.5 to focus in on most of the values. Point are ‘jittered’ to show variation within categories

 Fig. 6 shows how for most connectivity metrics the highest values *per unit area* – and therefore the most connectivity—are for the smallest buffer, and trends either remain level or decrease as buffer distance increases. Furthermore, obstacle counts *per unit area* also decrease with increasing buffer size. Given that connectivity, and barriers to it, scale in this way, the riparian zone is particularly important when considering issues of landscape management and consequent changes in connectivity.

### Patterns among connectivity metrics

**Fig. 7:**
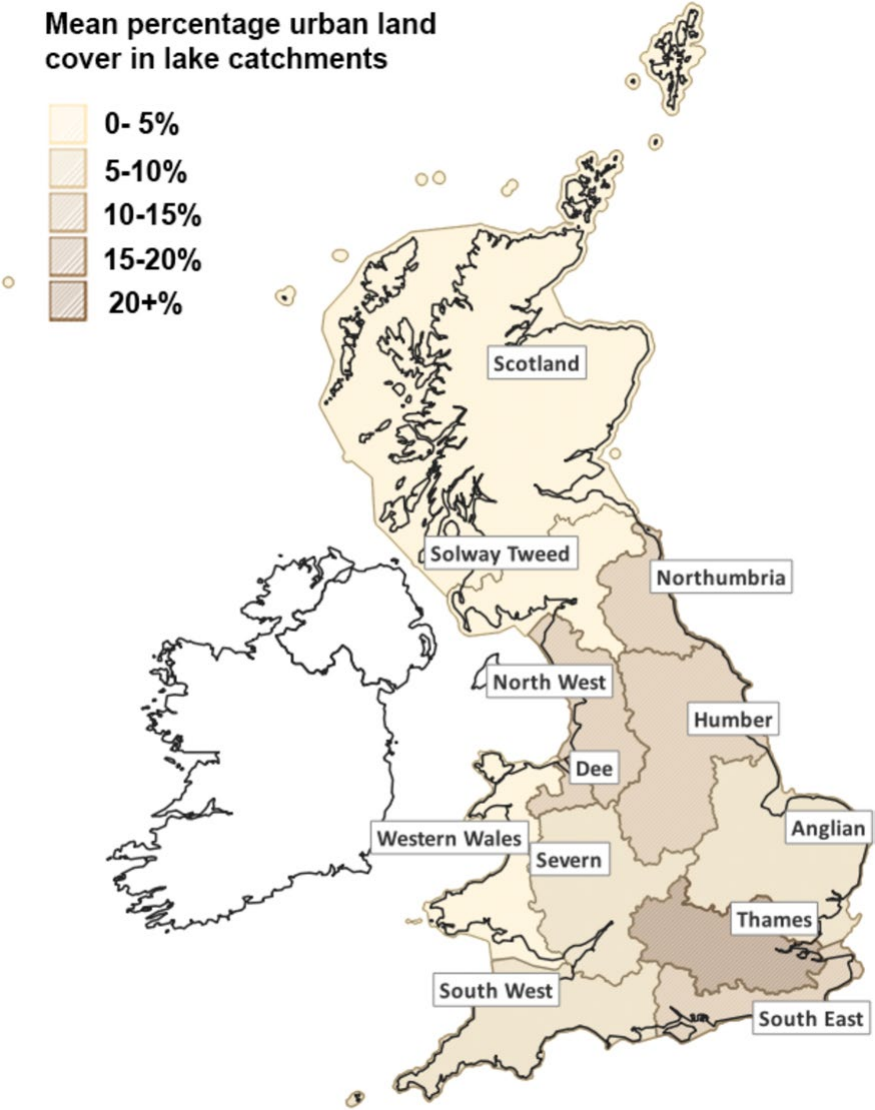
Water Framework Directive River Basin Districts for Great Britain, showing their mean percentage of urban land cover across the lake catchments

**Fig. 8:**
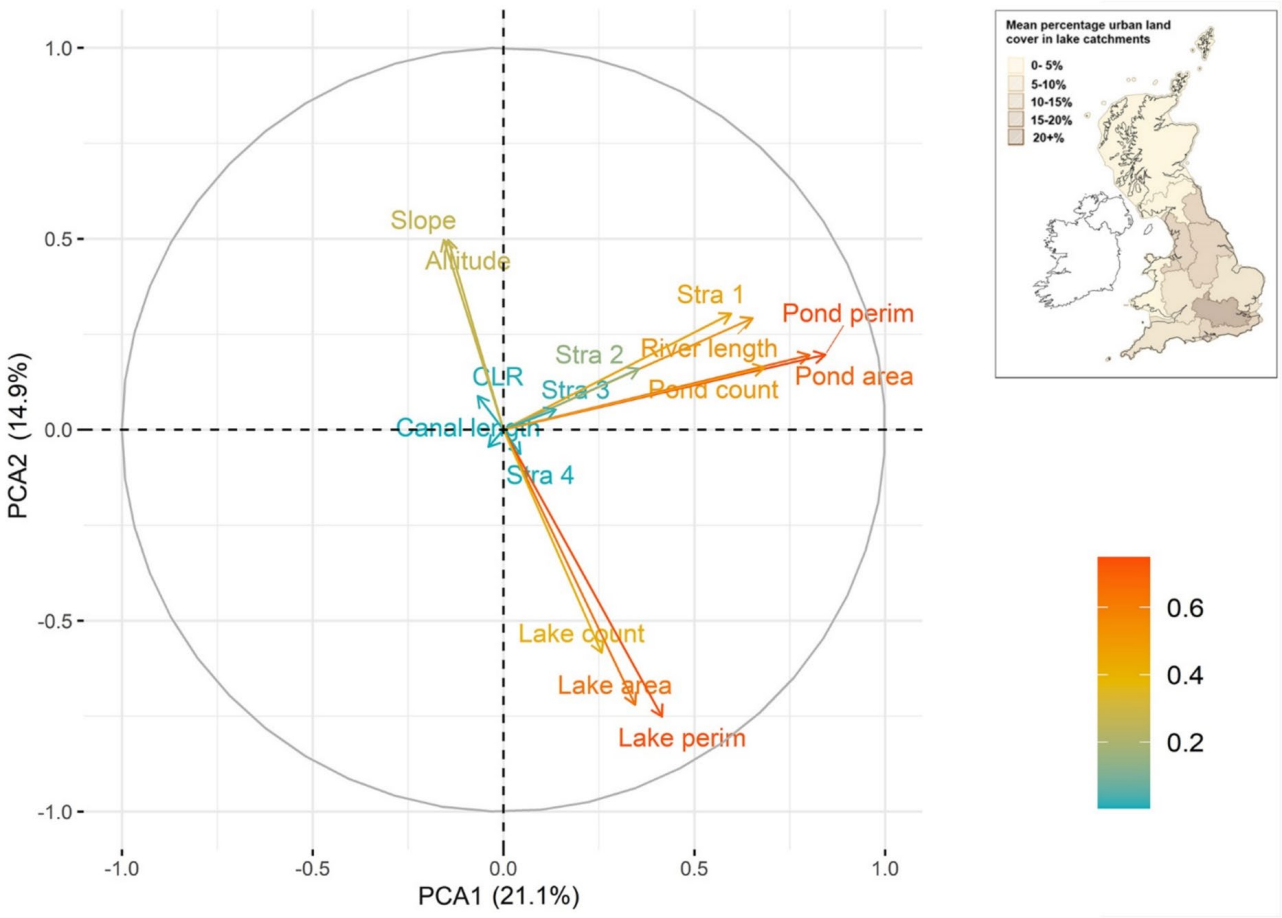
Principal Component Analysis for the connectivity metrics for all Great Britain lake (> 1 ha) catchments. In the colour legend, increasing scale from blue to red indicates better representation of the variable by the first two principal components. The inlay map shows Fig. 7

**Fig. 9:**
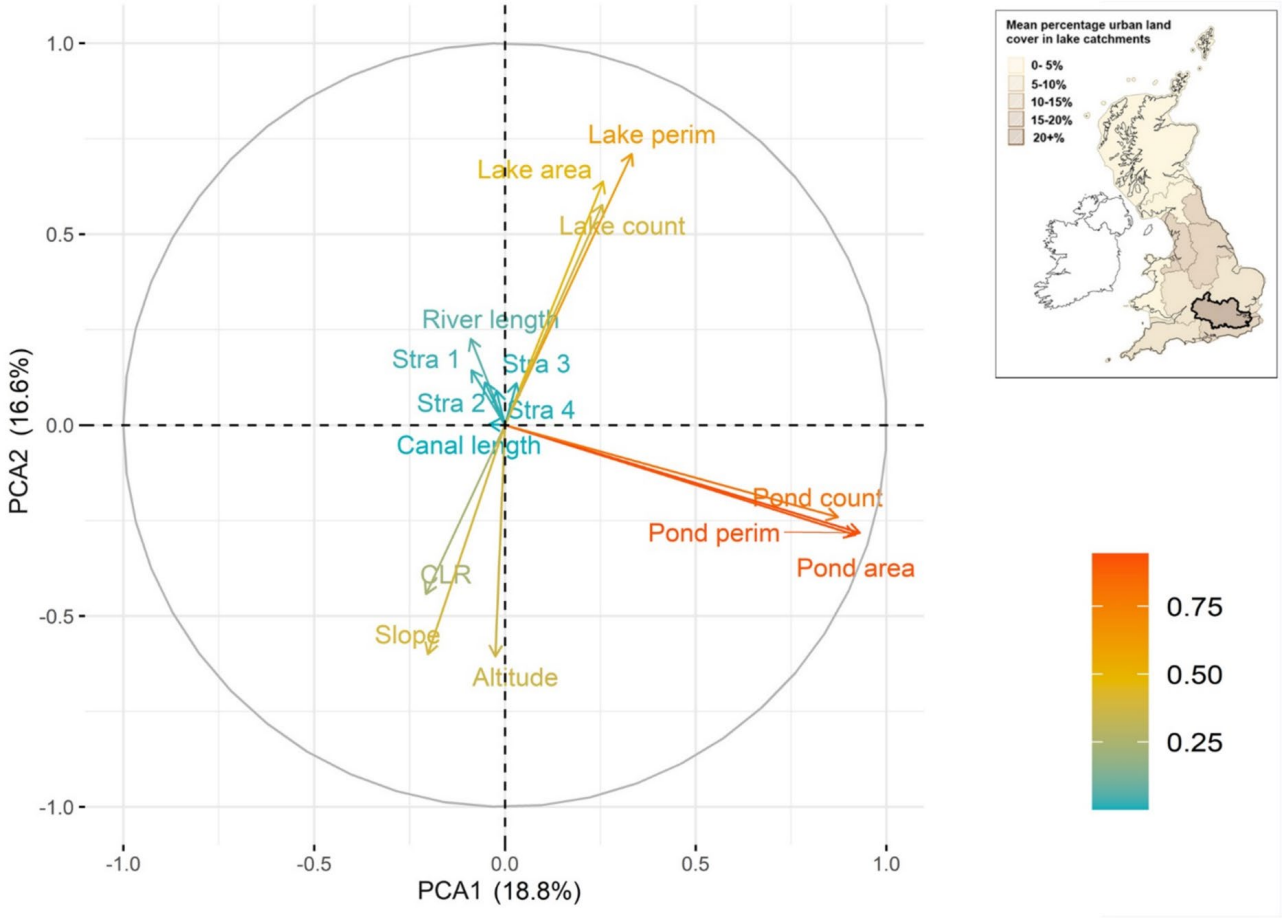
Principal Component Analysis for the connectivity metrics for lake (> 1 ha) catchments in the Thames River Basin District. In the colour legend, increasing scale from blue to red indicates better representation of the variable by the first two principal components. The inlay map shows Fig. 7, with the Thames River Basin District outlined in black

**Fig. 10:**
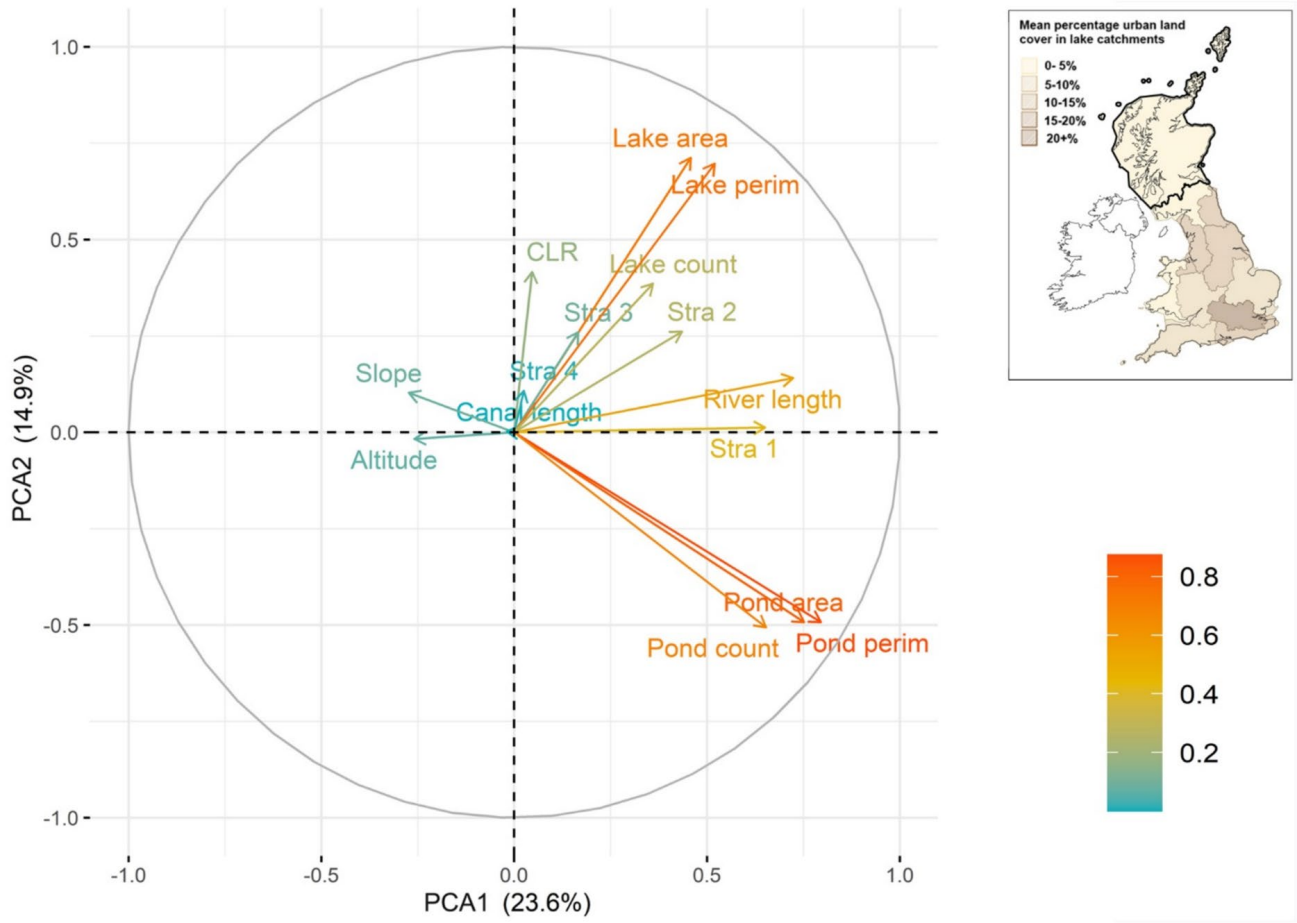
Principal Component Analysis for the connectivity metrics for lake (> 1 ha) catchments in the Scotland River Basin District. In the colour legend, increasing scale from blue to red indicates better representation of the variable by the first two principal components The inlay map shows Fig. 7, with the Scotland River Basin District outlined in black

 Table 3 shows a core set of metrics and stressors and how they differ across river basin districts. To exemplify this spatial variability in stressor intensity, mean percentage urban land cover for lake catchments within each RBD for Great Britain is shown in Fig. 7. Not only are the ranges in metric and stressor values considerable, but many regions vary greatly from the national average across multiple metrics. Maximum values for each metric are also spread around more than half the districts. To better understand these differences and the interaction between the metrics at different scales, we ran a PCA for all connectivity metrics across all lake catchments in Great Britain ( Fig. 8) as well as for individual RBDs, specifically those with the highest (Thames, Fig. 9) and lowest (Scotland, Fig. 10) levels of urbanisation in their catchments. At the national scale, the first two axes of the PCA accounted for 36.0% of the variability in connectivity metrics. For the Thames RBD this was 35.4%, and for the Scotland RBD 38.5% ( Table 4). These results show consistency in the percentage of variance explained, with, in each case, 2 principal components explaining about 40% of the variance, 3 principal components explaining about 50% and 6 principal components explaining about 70% of the variance in the hydrological connectivity metrics.

**Table 3:** A selection of mean connectivity metrics and stressors for lake (> 1 ha) catchments across Water Framework Directive River Basin Districts for Great Britain. River Basin Districts are sorted left to right in order of decreasing mean % urban area in the lake catchments. Highest values for each metric are in bold

Values	Thames	North West	Humber	South East	North- umbria	Dee	Anglian	Severn	South West	Western Wales	Solway Tweed	Scotland	*Great Britain (all)*
Catchment Count	582	494	688	172	146	54	646	366	199	287	313	**7048**	*10,995*
Mean catchment area (ha)	149.0	360.1	255.2	126.5	**530.5**	503.1	152.0	293.5	179.5	277.2	360.4	331.6	*303.5*
Mean catchment elevation (m)	58.0	173.3	120.6	43.0	181.7	228.7	33.6	183.1	118.8	**298.3**	194.3	192.9	*169.8*
Mean catchment LCM2007—urban %	**21.3**	13.1	11.7	11.0	10.5	10.2	9.0	7.3	6.6	4.6	1.2	0.6	*4.2*
Mean catchment LCM2007—agricultural %	44.9	37.6	51.0	46.3	41.4	37.1	**61.5**	54.1	47.5	18.5	29.8	4.9	*19.5*
Mean catchment obstacle count (#/100 ha)	**0.4**	**0.4**	0.3	0.2	0.1	0.1	0.0	0.1	0.1	0.1	0.0	0.0	*0.1*
Mean catchment lake area %	**3.5**	0.7	2.0	1.2	0.5	1.9	2.2	0.6	1.2	0.5	0.3	1.7	*1.7*
Mean catchment pond count (#/100 ha)	0.7	0.3	1.2	0.3	0.4	0.4	1.5	0.9	**2.1**	0.4	0.3	3.2	*2.4*
Mean catchment rivers length (m/100 ha)	840.4	706.3	785.9	**1451.6**	978.0	458.3	521.7	566.9	631.9	933.6	1058.0	1374.8	*1160.0*

**Table 4:** Cumulative % variance (to 2 decimal places) for the first 6 principal components (PCs) for all catchments in the Great Britain dataset and each of the Scotland and Thames River Basin Districts

Cumulative % variance for each catchment	PC1	PC2	PC3	PC4	PC5	PC6
All Great Britain	21.06	35.98	48.95	58.07	64.91	71.60
Scotland River Basin District	23.62	38.51	51.66	59.98	67.52	74.43
Thames River Basin District	18.77	35.38	49.26	59.13	66.01	72.81

All three PCAs showed that metrics representing lakes (area, perimeter, count) were correlated. A similar result was found for metrics representing ponds (area, perimeter, count). The vectors for metrics representing lakes were near-perpendicular to those for ponds in the national, Thames and Scotland PCAs, showing that these lake and pond metrics were uncorrelated with each other. This suggests that lake and pond connectivity metrics in a landscape vary independently and provide different information on overall lake connectedness (i.e. pond count in a catchment does not necessarily increase where lake count in a catchment does).

The national and Thames PCAs showed negative correlations between catchment mean slope and lake elevation, and the lake metrics. Specifically, more upland areas are associated with fewer lakes, less lake area, and less lake perimeter habitat. In the Scotland RBD, catchment mean slope and lake elevation were instead negatively correlated to the pond metrics and were poorly correlated to the lake metrics. This suggests that upland areas were associated with fewer ponds in the Scotland RBD but had little relationship with lake metrics.

At a national-scale, spatial variations in most river metrics (overall length, and Strahler 1, 2 and 3 segment length) were well correlated with each other, and to the pond metrics. That Strahler 4 + segment length (the main large river channel in the United Kingdom) was less well correlated with the other metrics is likely to be due to there generally being few Strahler 4 + segments in catchments nationally. River length was consistently correlated with Strahler 1, indicating that total river length in each River Basin District was predominantly due to the length of headwater streams. At the scale of the Thames RBD, the river length metrics were less well correlated with each other and were not well represented (short vectors) by the first two PCs that explain most variability in the dataset. For the Scotland RBD, two of the river length metrics (larger downstream channels of Strahler 3 and 4) were positively correlated with the lake metrics, whereas Strahler 1 (headwater streams) and total river length were not, but were correlated with each other. The differing directions of the Strahler segment length vectors suggested that spatial variations in Strahler lengths were less well correlated in this RBD, whereas nationally, and for the Thames RBD, river length of any Strahler type (1 to 3) could be well represented by a simple river length metric.

### Variability in connectivity metrics across river basin districts

For simplicity, and based on the PCA results, in the rest of this paper we have selected pond count, lake area and river length as a core set of connectivity metrics to explore further. The average values of these three core connectivity metrics for lake catchments within each RBD are shown in Table 3 alongside average lake and catchment characteristics (lake elevation and mean catchment slope) and three landscape characteristics that act as proxies for the level of anthropogenic stress on freshwater biodiversity (% agricultural and % urban land in catchments and counts of river obstacles).

The twelve River Basin Districts in Great Britain vary in mean catchment area, elevation and slope ( Table 3) with Western Wales having the highest altitude lakes and the Thames and south-east England the lowest. Unexpectedly, the greatest % lake area in catchments was found in the Thames RBD, the most urbanised RBD which is explained by the fact that it has many large reservoirs supplying water to this densely populated region. Similarly, the lowest % lake area was found in some of the least urbanised regions (Solway-Tweed and Western Wales). Pond count was highest in the agricultural dominated Anglian RBD and lowest in the upland North West RBD. River length (density) was highest in the South-East and second highest in Scotland – two very different RBDs, but the SE having many fewer lakes. River length tended to be lower in the more agriculturally dominated catchments (Anglian, Severn).

**Fig. 11:**
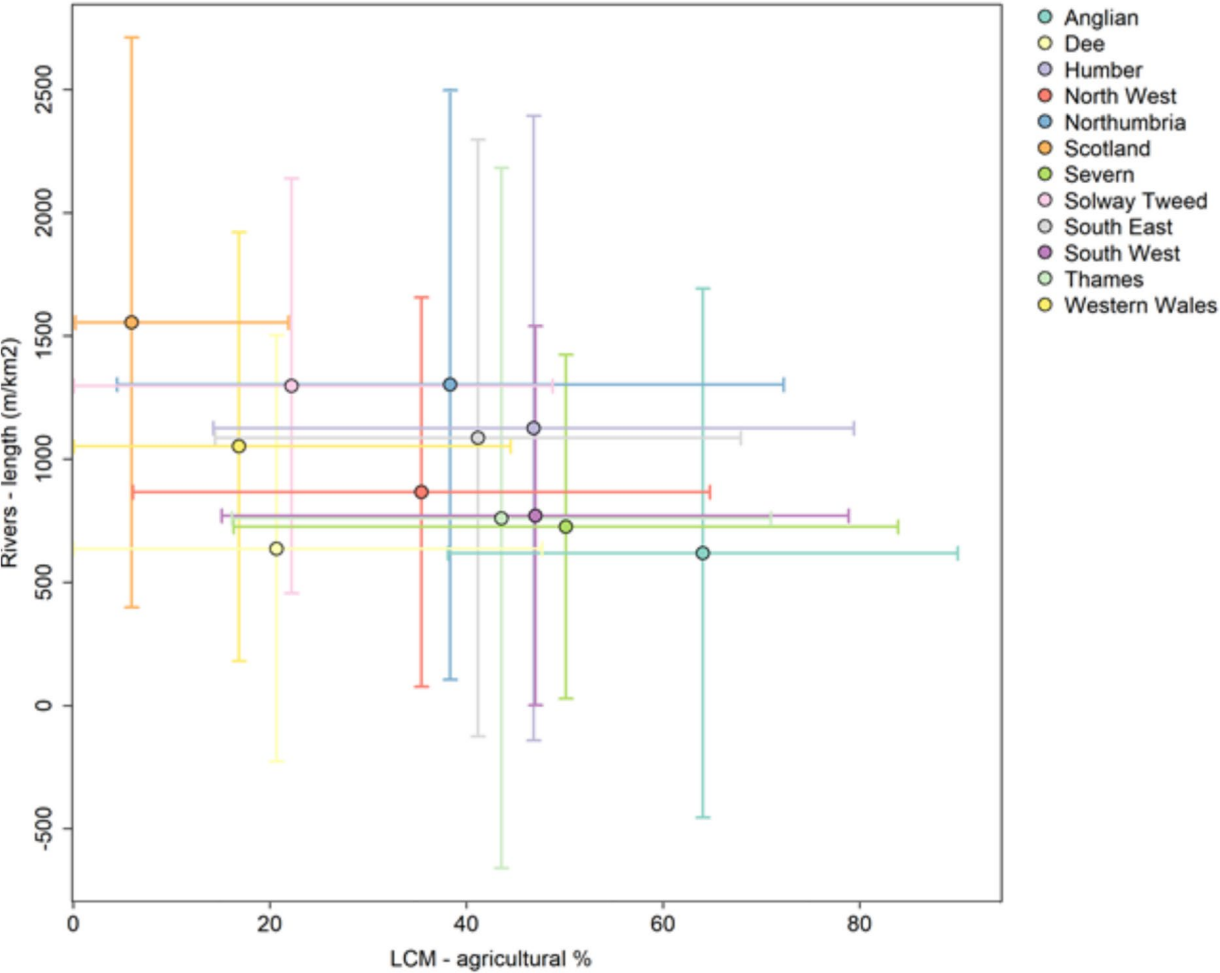
Scatter plot showing the relationship between % agricultural land vs river length *per unit area* across the twelve River Basin Districts. Points are the mean values for each River Basin District and error bar endpoints show the mean values ± standard deviation

**Fig. 12:**
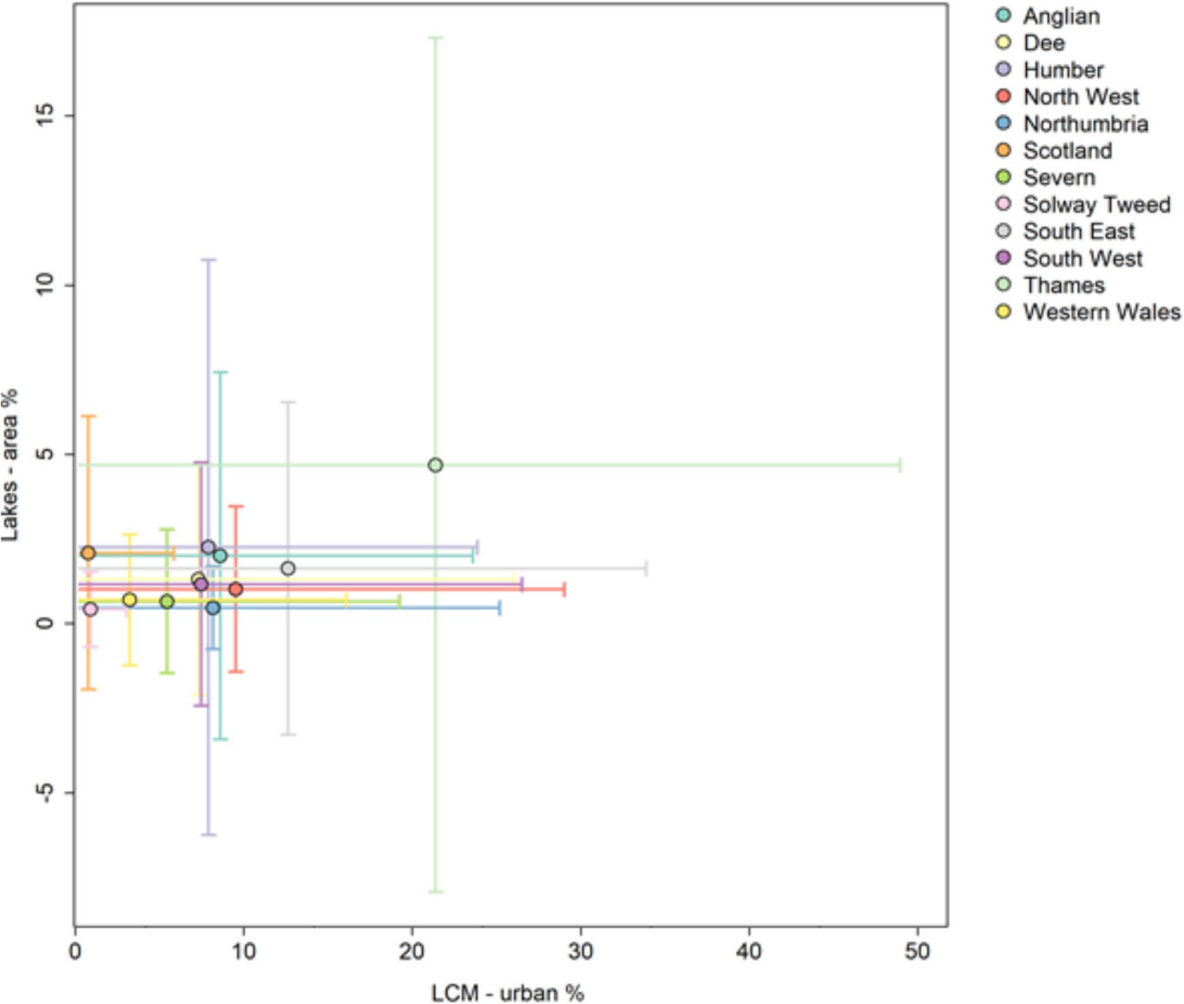
Scatter plot showing the relationship between % urban land and % lake area across the twelve River Basin Districts. Points are the mean values for each River Basin District and error bar endpoints show the mean values ± standard deviation

Further exploratory analysis evaluated how the average values of these three core connectivity metrics in each RBD correlated with metrics of landscape “stress”. The strongest relationship was observed between mean % agricultural land and mean river length in lake catchments ( Fig. 11) which indicates that lakes with increasing % agricultural land generally had a lower density of rivers in their catchments. The second strongest relationship was between % urban land and % lake area in lake catchments ( Fig. 12) which suggest that lakes with increasing % urban land generally had increasing % lake area in their catchments. However, Fig. 12 shows this positive relationship was largely influenced by the most urbanised Thames RBD. It is again worth noting that ‘lakes’ include lakes and reservoirs, and the latter are well represented in the Thames RBD, so are likely having an influence.

### Core connectivity metrics

In summary, the PCAs show us that, because of the consistently high correlations within a cluster of connectivity metrics (e.g. all three pond metrics), there is no need to include every metric when analysing influences of connectivity in freshwater systems, and one metric from each cluster can be used to represent the (minimum) different components of freshwater connectivity in lake catchments. Broadly speaking, these distinct clusters represent the amount of river, pond and lake habitats within the focal lake catchment.

Therefore, the core connectivity metrics were defined as lake area, river length and pond count. These choices were based on a mixture of practicality, flexibility and explanatory power, considering not just the national scale ( Table 2; Fig. 8), but the variation shown across river basin districts ( Table 3; Fig. 9; Fig. 10).

## Discussion

In this study, we quantified connectivity of lakes in Great Britain to other freshwater habitats based upon metrics of the abundance and extent of several waterbody types within either hydrological catchments or spatially concentric buffer zones radiating out from each focal lake site. Each metric potentially provides different information on connectivity and can, therefore, be utilised for studies researching specific connectivity aspects. However, we show that there were strong correlations among subsets of metrics associated with ecosystem type (lake, river or pond metrics) and that these could help to simplify future analysis on freshwater connectivity.

### Hydrological connectivity

Around a third of lakes appeared isolated from surface hydrology, at least with no major in- or out- flows visible at 1:50,000 scale (Moore et al. 1994). Around a third of all lakes were drainage lakes with inflowing and outflowing rivers, but with no connected lake upstream, while a quarter were drainage lakes with another lake upstream in their catchment. It is worth noting that due to the scale of the river network data, these figures do not consider connectivity at finer spatial scales (flow/drainage paths at 1:50,000 scale are equivalent to a 25 m grid resolution using Tobler’s rule).

The size of a waterbody influenced connectivity. The largest lakes in our study (> 10 ha) were the most connected hydrologically to other upstream lakes, as they were predominantly in larger catchments. Isolated systems tended to be smaller, with ponds being hydrologically isolated, with no in- or outflow. We also found that “sink systems” were rare, most probably due to the wet, temperate climate and relatively short distances to the sea in the United Kingdom; sink systems are more typical of arid, continental regions (e.g. Australia, Mongolia, etc.) (Jellison et al. 2008). Although isolated and drainage lakes were clustered in the north and west of Scotland, they and most other lake connectivity types had a broad distribution, suggesting that geological history is more important than landscape features (e.g. land use) for lake distribution. Due to the scale of the input data and a lack of groundwater connectivity data, these figures for sink systems and isolated lakes will include lakes that have only local connectivity and groundwater-only-fed lakes, such as in the Cheshire-Shropshire meres (Carvalho & Moss 1999). Overall, within regions or countries, we found a range of lake connectivity types and that connectivity was mostly related to waterbody size and climatic region. This likely reflects the creation of similar lake types by common geological processes, regardless of current or historic land use.

### Landscape connectivity

The effect of land use on fresh waters is often scale-dependent, with its greatest impact occurring at close proximity (Amoros & Bornette 2002), although impact will be modified by drainage density. Pedersen et al. (2006) used buffers at varying distances from lake shores to examine the effect of land use on the occurrence of macrophyte species. Their results showed that land use within a buffer zone of less than 3 km exerted a stronger effect on the occurrence of the macrophyte *Littorella uniflora* than land-use at larger spatial scales. Others have also shown that the proportion of managed land within the immediate vicinity of a lake exerts a significantly greater influence on macrophyte richness than at the broader catchment scale (Steffan-Dewenter et al. 2002; Sun et al. 2018). Similarly, the impact of land-use on the movement of materials and nutrients is likely to have a diminishing effect further from shorelines or riparian zones. This is one key reason that riparian buffer zones and shorelines are often the target area for landscape management measures to reduce the impacts of pollution on water quality and ecological health (Broadmeadow & Nisbet 2004). The variation across spatial scales in this analysis was very high, with standard deviations often higher than mean values. This highlights that the scale of study is important in terms of metric selection, as well as the ecological context being studied.

### Correlation/redundancy in connectivity metrics

In general, our analyses show strong correlations among metrics of pond, lake and river connectivity classes that support the selection of any metric within each class as a representative metric. However, we also found that the relationships between connectivity metrics differ at different geographical scales (Great Britain vs RBDs) and land use categories, suggesting that the choice of connectivity metrics can be tailored depending on the scale and location of study. This may be important for future ecological studies evaluating the impact of connectivity on biodiversity or ecosystem functioning. This (and the individual catchment PCA analysis) illustrates that a simplification in use of connectivity metrics can usefully be achieved but that it may still be required to investigate additional connectivity metrics, and that to understand and quantify freshwater connectivity at the sub-national scale, a varied set of connectivity metrics are required for impact assessment.

It was broadly shown that freshwater spatial connectivity cannot be summarised as a single metric (e.g. total freshwater in an area) that would be equally relevant to a wide range of organisms with different dispersal traits. To evaluate the connectivity of a lake with its landscape, a multi-metric approach is required that recognises the variation in lake, pond and river distribution within that landscape. It is therefore important to determine the metrics that are most appropriate to a particular ecological phenomenon or organism group, and which can be measured with the highest degree of certainty for each habitat. As such, hypothesis-driven metric selection is essential for the management of fresh waters considering future land use and climate changes that may affect ecosystem state, processes and services via connectivity-mediated mechanisms.

### Defining core connectivity metrics

The three main clusters of metrics identified from the PCAs were further reduced to three core connectivity metrics – lake area, pond count and river length. This is presented as the minimum required for understanding the effect of connectivity on a lake or its biota—in terms of its hydrological catchment, riparian zone or landscape buffers. The choices made to reduce these connectivity metrics to a core set balanced statistical explanatory power, range of use across studies and potential availability of data. Therefore, these three metrics will have maximum usability for comparative global studies and for areas where data availability is scarce.

Lake area is known to be an important driver of freshwater species richness (Brucet et al. 2013; Dodson et al. 2000) so may be a useful connectivity metric to apply in ecological studies. In general, lake polygons are well-defined, and all the lake metrics can be calculated easily from suitable global datasets (e.g. Sikder et al. 2023). River length was clearly the most variable of the river metrics between catchments in the dataset (longest vector of the river metrics in the PCAs). This is also likely to support more sensitive analyses with respect to variability among rivers, as well as being a useful representative metric to explore variation in ecological responses to availability of river habitat in a catchment. For ponds, there may be more uncertainty in the estimates of pond area and perimeter in catchments at the mapping scale used in this study. Pond count, more so than the other metrics, can be changed in a landscape through in-filling, terrestrialisation and creation, with the latter also being actively promoted as a nature-based solution for restoring biodiversity and water management (e.g. https://ponderful.eu/). Lake and pond connectivity metrics were shown in the PCAs to be largely uncorrelated with each other, which is likely primarily related to the way these different waterbodies are created, with lakes developing from geological processes but many ponds being created by humans, meaning their location is not constrained by the landscape in the same manner. There is also uncertainty in the number, and spatial variation of, ponds in this study due to a lack of data availability for the smallest waterbodies.

Finally, the core (minimum) set of connectivity metrics presented should not be seen as comprehensive for any study analysing hydrological connectivity, as explained in the discussion above.

### Modelling different biodiversity responses

In this study, we have examined a small representative subset of metrics that are relevant to general management of freshwater biodiversity across large landscapes. Metric selection should, however, be tailored to the questions being answered. For example, ecological studies of dragonfly or shoreline plant biodiversity may preferentially focus on metrics of lake perimeter or river length, rather than count or area, whereas it may be more relevant to select area-based metrics in studies of roosting wetland birds.

Long-distance migratory species, such as Atlantic salmon (*Salmo salar*), European eel (*Anguilla anguilla*), sea trout (*Salmo trutta*) and sea lamprey (*Petromyzon marinus*) have complex life cycles that depend greatly upon upstream–downstream connectedness from headwaters to the sea. Mean catchment obstacle count was clearly correlated with percentage urban areas in lake catchments. Fish, however, can be impacted by just one barrier upstream and downstream (Coté et al*.* 2009; Horreo et al. 2011), so more nuanced metrics on the type of barrier and its permeability to different fish species are really needed for future studies on barrier impacts.

## Conclusions

To guide further research into assessing connectivity with respect to lakes and the freshwater habitats in the surrounding landscape or their hydrological catchments:

- Principal Component Analysis shows that, because of the consistent high correlations within clusters of connectivity metrics, there is no need to include every metric when analysing influences of connectivity in freshwater systems.
- A minimum set of connectivity metrics should include river length, pond count and lake area, all calculated *per unit area* for the catchment / buffer zone.
- The size of a waterbody generally influences connectivity. The largest lakes in our study (> 10 ha) were the most connected hydrologically to other upstream lakes.
- Overall, we found that connectivity was mostly related to waterbody size and climatic region. This likely reflects the creation of similar lake types by common geological processes, regardless of current or historic land use.
- The effect of land use on fresh waters is often scale-dependent, with its greatest impact occurring at close proximity, although impact is modified by drainage density.
- At smaller scales, the choice of connectivity metrics should be tailored depending on the location, theme and context of study. It is therefore important to determine the metrics that are most appropriate to a particular ecological phenomenon or organism group, and which can be measured with the highest degree of certainty for each habitat. As such, hypothesis-driven metric selection is still preferable, if possible.

## Data Availability

The core set of metrics from this study are available on the UK Lakes Portal: https://uklakes.ceh.ac.uk/
